# Detecting colorectal cancer using genetic and epigenetic biomarkers: screening and diagnosis

**DOI:** 10.25122/jml-2023-0269

**Published:** 2024-01

**Authors:** Yudith Annisa Ayu Rezkitha, Nur Syahadati Retno Panenggak, Maria Inge Lusida, Raissa Virgy Rianda, Isna Mahmudah, Aditya Doni Pradana, Tomohisa Uchida, Muhammad Miftahussurur

**Affiliations:** 1 Doctoral Program of Medical Science, Faculty of Medicine, Universitas Airlangga, Surabaya, Indonesia; 2 Helicobacter pylori and Microbiota Study Group, Institute of Tropical Disease, Universitas Airlangga, Surabaya, Indonesia; 3 Institute of Tropical Disease, Indonesia-Japan Collaborative Research Center for Emerging and Re-Emerging Infectious Diseases, Universitas Airlangga, Surabaya, Indonesia; 4 Department of Child Health, Faculty of Medicine, Universitas Airlangga, Surabaya, Indonesia; 5 Department of Internal Medicine, Faculty of Medicine, Universitas Airlangga, Surabaya, Indonesia; 6 Department of Emergency Services, Kendal Islamic Hospital, Kendal, Indonesia; 7 Department of Cardiology and Vascular Medicine, Faculty of Medicine, Public Health and Nursing, Gadjah Mada University, Yogyakarta, Indonesia; 8 Department of Molecular Pathology, Faculty of Medicine, Oita University, Yufu, Japan; 9 Division of Gastroentero-Hepatology, Department of Internal Medicine, Faculty of Medicine-Dr Soetomo Teaching Hospital, Universitas Airlangga, Surabaya, Indonesia

**Keywords:** colorectal cancer, cancer, genetic biomarkers, epigenetic biomarkers, diagnostic biomarkers

## Abstract

Colorectal cancer (CRC) is one of the most frequent types of cancer, with high incidence rates and mortality globally. The extended timeframe for developing CRC allows for the potential screening and early identification of the disease. Furthermore, studies have shown that survival rates for patients with cancer are increased when diagnoses are made at earlier stages. Recent research suggests that the development of CRC, including its precancerous lesion, is influenced not only by genetic factors but also by epigenetic variables. Studies suggest epigenetics plays a significant role in cancer development, particularly CRC. While this approach is still in its early stages and faces challenges due to the variability of CRC, it shows promise as a potential method for understanding and addressing the disease. This review examined the current evidence supporting genetic and epigenetic biomarkers for screening and diagnosis. In addition, we also discussed the feasibility of translating these methodologies into clinical settings. Several markers show promising potential, including the methylation of vimentin (*VIM*), syndecan-2 (*SDC2*), and septin 9 (*SEPT9*). However, their application as screening and diagnostic tools, particularly for early-stage CRC, has not been fully optimized, and their effectiveness needs validation in large, multi-center patient populations. Extensive trials and further investigation are required to translate genetic and epigenetic biomarkers into practical clinical use. biomarkers, diagnostic biomarkers

## INTRODUCTION

**Figure 1:**
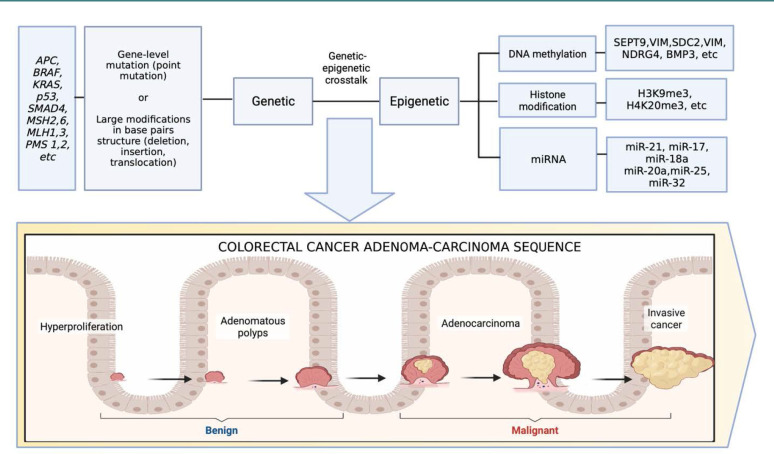
Proposed genetic and epigenetic biomarkers for potential use in CRC screening and diagnostic. Adapted from “Benign and malignant colorectal cancer”, by BioRender.com (2022). Retrieved from https://app.biorender.com/biorender-templates.

The estimated number of new cases of colorectal cancer (CRC) in 2018 was 1.85 million, representing about 10% of all cancers worldwide [1,2]. In 2018, 880,792 (9.2%) deaths were estimated to be attributable to CRC [2]. Recent data has revealed a concerning trend in the incidence rate of CRC, indicating a global rise from 1990 to 2019 [3,4]. CRC typically develops from a precancerous lesion known as an adenoma through a multi-step process termed the 'adenoma-carcinoma sequence'. This transformation can span 10 to 15 years [5]. This extended duration offers a crucial window for screening and early diagnosis of the precancerous lesion before its transformation into cancer (Figure 1) [6]. The improvement of screening programs could increase detection and decrease the incidence rate of advanced cancer, which also improves overall cancer management, prognosis, and death rates related to CRC [7,8]. Moreover, when detected in the early phase of the disease and combined with prompt therapy, the 5-year survival rate has a better outcome of more than 90% in the localized stage compared with 10% in patients with metastasis [9]. As a result, it is essential to develop a procedure that can increase the number of people who undergo screening, is easy to implement on a massive scale, and has high levels of sensitivity and specificity.

Screening is recommended for individuals with a moderate risk of CRC, typically between 50 and 75 [10]. Currently, there are numerous ways for detecting colorectal cancer, including invasive methods such as flexible sigmoidoscopy and colonoscopy and less invasive approaches such as guaiac fecal occult blood test (gFOBT) and fecal immunochemical test (FIT) [11]. Despite its reliability, colonoscopy is less favored due to higher costs, discomfort, potential complications, and lower patient compliance [12]. Studies indicate a preference among patients for less invasive screening methods [13], underscoring the need for an ideal screening approach that balances invasiveness with high specificity and sensitivity.

One of the well-established pathways in CRC begins with a mutation in the adenomatous polyposis coli (*APC*) gene [14]. After this event, mutations occur in the rat sarcoma viral oncogene homolog (*RAS*) and tumor protein 53 (*TP53*) genes and other genes [15]. In addition, extensive research has demonstrated the significance of genetic and epigenetic changes in CRC carcinogenesis [16]. Based on this knowledge, many studies have recognized genetic and epigenetic alterations as potential new biomarkers for use in screening, diagnosis, and even predictive biomarkers of therapy response throughout the past decade [17-19]. Their detection is possible in various biological samples, such as tissue, blood, stool, and urine. The goal of this review was to compile genetic and epigenetic markers with potential of early detection and diagnosis both presently and in the near future.

### The genetic and epigenetic mechanism in CRC

Genetic and epigenetic changes were initially identified as independent CRC pathways. However, recent research suggests an interaction between these two CRC carcinogenesis pathways (Figure 1) [20]. Genetic mutations modify epigenetic regulation, allowing genomic instability and mutagenesis [21]. The epigenetic factors dysregulating genes involved in DNA mismatch repair (MMR) often result in genomic instability and dysregulation of genes involved in carcinogenesis (oncogenes and tumor suppressor genes) [22,23]. CRC is a multifactorial disease, and numerous pathways have been studied. Among these, three prominent pathways have been widely reported. The first two are usually referred to as traditional pathways, namely chromosomal instability (CIN) and microsatellite instability (MSI) [24,25]. The other pathway is the CpG island methylator phenotype (CIMP), also called the serrated pathway of CRC [24–26]. In addition, some of these pathways might be complexly interconnected. Microsatellite instability and chromosomal instability are commonly viewed as distinct mechanisms through which sporadic CRC develops, and it has been suggested that CIMP may be behind the development of MSI and/or CIN [27]. CIN is often detected in the majority of CRC cases [28]. CIN is reportedly characterized by aneuploidy and allelic loss at chromosome 18q (18q LOH) [29,30]. It is also characterized by *KRAS* activation, a well-known oncogene in CRC, as well as mutations that inactivate tumor-suppressor genes such as *APC* and *TP53* [29,31]. MSI is caused by a reduction in DNA mismatch repair activity, defined by length changes within simple repeated sequences known as microsatellites. This event is reported in 15% of CRC cases [32,33]. The last pathway of the three is CIMP, a subset of CRC that can be identified by the extensive methylation of promoter CpG island sites surrounding the promoting regions of several genes [34,35].

### The role of genetics in CRC

CRC predominantly develops through three distinct patterns: sporadic, inherited, and familial [36]. The majority of cases (75–80%) are sporadic, around 25% are familial with a family history of the disease but no associated germline mutation, and hereditary cases comprise approximately 10% [36,37]. Genetic alterations in cancer are characterized by small changes in nucleotide sequences or gene-level mutation (point mutation) and significant modifications in base pairs structure (deletions, insertions, and translocations) [38]. The carcinogenesis process typically involves dysregulation of oncogenes, tumor suppressor genes, and DNA repair genes [25]. Multiple pathogenic germline variants have been linked to a predisposition to hereditary CRC or polyps [39]. There are a number of hereditary disorders that have a strong correlation with the development of polyps in the colon. These conditions include but are not limited to, familial adenomatous polyposis (FAP), which is closely related to alteration of the *APC* gene, MUTYH-associated polyposis (MAP), caused by biallelic *MUTYH* mutations, polymerase proofreading–associated polyposis (PPAP) associated with mutations in the *POLE* or *POLD1* genes [40,41].

### Genetic biomarkers for screening and diagnosis of CRC

The Kirsten rat sarcoma (*KRAS*) gene is one of the oncogenes most frequently mutated in CRC, with mutations found in approximately 35-45% of all CRC cases [42]. This mutation is often linked to tumors in the right colon phenotype, and roughly 85% of all *KRAS* mutations occur in one of three primary hotspots (codons 12, 13, and 61) [43]. The presence of *KRAS* mutations has been recognized for its prognostic significance and its ability to predict the efficacy of therapeutic interventions [44]. Patients with *KRAS* mutation generally have poorer prognosis than patients without such mutation [45,46]. Inappropriate activation of the *KRAS* pathway disrupts the upstream signal control of *KRAS*, which ultimately causes resistance to receptor tyrosine kinase (RTK) inhibitors [47]. Consequently, testing for *KRAS* mutations is recommended before administering anti-epidermal growth factor receptor (EGFR) therapy [44]. Furthermore, *KRAS* mutations promote liver metastasis by upregulating the expression of IGF-1R through a new mechanism involving MEK-SP1-DNMT1-miR-137 [48]. Another gene involved in the RAS/RAF/MEK/ERK signaling pathway is *BRAG*, which, alongside *KRAS*, is a component of this signaling [49]. This pathway is necessary for proper cell proliferation, differentiation, survival, and apoptosis [50]. According to some reports, *BRAF* mutations are associated with a poor prognosis and occur in approximately 10% of CRC cases [51]. The unique characteristics of *BRAF* mutations suggest they may influence the therapeutic response, though further research is needed to clarify their specific impact on treatment outcomes [52,53].

The *PIK3CA* gene is another frequently mutated gene in CRC, accounting for 10–20% of patients with CRC [54,55]. Mutations in *PIK3CA* are often found in cancers located in the proximal colon and are associated with a high level of CpG island methylator phenotype (CIMP) [51]. Moreover, *PIKCA* mutations correlate with mucinous differentiation, *KRAS* mutations, and microsatellite instability [51,56]. Both in vivo and in vitro studies revealed that mutations in *PIK3CA* were related to resistance to first-line chemotherapy treatment [57]. In addition to *PIK3CA* and *KRAS*, the *TP53* gene was reported to be altered in 43% of CRC cases, and the remaining cancers frequently have reduced p53 activity due to mutations in other genes regulating p53 [58]. Under conditions of cellular stress, the protein TP53 performs the role of a transcription factor and is responsible for the initiation of cell cycle arrest, senescence, and apoptosis [59]. A meta-analysis reported that the diagnostic value of serum p53 showed a pooled sensitivity of 0.19 (95% CI, 0.18–0.21) and a pooled specificity of 0.93 (95% CI, 0.92–0.94) [60].

Allelic loss on chromosome 18q is an additional mutation that significantly impacts CRC, detected in up to 70% of primary CRC cases, especially in the late stages [16]. Studies have also associated 18q loss of heterozygosity (LOH) with poorer prognosis, underlining its clinical relevance [61]. The regions affected by LOH on chromosome 18q are believed to inactivate three distinct genes in CRC, including *DCC, DPC4/SMAD4*, and *SMAD2* [62]. In addition, LOH has also been linked to liver metastasis [63]. Genetic testing and counseling are beneficial for persons at high risk of familial or inherited CRC, especially first-degree relatives, as they can identify susceptibility to inheriting this form of cancer. However, genetic testing should focus on intermediate and high-risk patients instead of population-based screening techniques [64].

For instance, testing for mismatch repair deficiency is advised for screening for Lynch syndrome [65], the most common form of hereditary CRC, which accounts for about 10% of all CRC cases and is associated with mutations in mismatch repair genes [66]. Understanding genetic predisposition is crucial for colorectal cancer screening and early diagnosis. Advancements in this field are key to narrowing the gap between research and clinical practice.

### Epigenetics as emerging biomarkers in CRC

In recent years, the intersection of cancer research and epigenetics has begun to attract significant attention. Epigenetics refers to heritable modifications in gene expression that do not involve alterations to the DNA sequence [67]. Histone modifications, DNA methylation, remodeling of the chromatin, and non-coding RNA (ncRNA), particularly miRNA, are epigenetics alterations that are believed to be essential in CRC development and progression [68–70]. Studies have shown that as CRC progresses from early-stage adenomas to advanced stages, a considerable number of aberrant methylated genes appear to increase drastically, with different frequencies characterizing each progression step [71]. This is one of the many reasons epigenetics are now emerging as biomarkers for diagnosis and screening and prognostication and response to therapy [72,73]. Their presence can be detected in less invasive blood, stool, and urine samples, offering a less invasive alternative to traditional screening methods like colonoscopy [74]. Furthermore, there is a growing consensus that epigenetic changes can occur early in carcinogenesis, manifesting more frequently than genetic alterations [75].

### DNA methylation markers are one of the most promising CRC markers

DNA methylation involves the addition of a methyl group to the C-5 position of the cytosine ring within DNA facilitated by DNA methyltransferases [70], which can modify the activity of a DNA segment without altering its sequence [68]. This epigenetic mechanism is implicated in the regulation of hundreds of genes in CRC, making DNA methylation an intriguing biomarker candidate [76]. In addition, methylation of oncogenes and tumor suppressor genes may already be present in the early phases of the transformation into a malignant state [77].

During the onset of cancer, hypermethylation in the promoter region may result in the inactivation of tumor-suppressor genes, whereas global hypomethylation is linked to genomic instability and chromosomal abnormalities [70]. While hypomethylation is a gradually early event in tumor progression, hypermethylation accumulates in more advanced stages [69,78]. Blood and stool-based CRC DNA methylation indicators have exhibited sensitivities between 90-95% and specificities between 85-95% [79]. The FDA has currently approved two methylation-based diagnostic biomarkers for CRC: *SEPT9* and the combination of bone morphogenetic protein 3 (*BMP3*) and N-Myc downstream-regulated gene 4 (*NDRG4*) [77,80]. *SEPT9* has emerged as a helpful screening marker in the blood samples of patients, allowing the detection of CRC at various stages and colonic sites [81]. *SEPT9* methylation is one of the most popular markers for CRC compared to any other single methylated marker.

Two commercially available *SEPT9* blood tests for CRC screening are already in clinical use. These include ColoVantage (sensitivity of 90%) [82] and Epi proColon 2.0 (sensitivity of 66–81% and specificity of 96–9%) [83–85]. Carcinoembryonic Antigen (CEA) is one of the biomarkers used in CRC, and a study showed that *SEPT9* is better at detecting CRC than CEA. *SEPT9* has a sensitivity of 75.6%, while CEA only has a sensitivity of 47.7% [80]. Numerous researchers have validated *SEPT9* as a significant marker for the early detection of CRC, demonstrating its superiority over other markers, such as CEA, when used as a single marker [86]. The effectiveness of *SEPT9* methylation as a CRC detection marker varies with the stage of the tumor, showing an increased positive rate in correlation with advancing tumor stages [87]. Combining *SEPT9* methylation with CEA testing enhances sensitivity, offering a more effective approach for early CRC detection [88].

The FDA has also approved Cologuard, a commercially available stool-based test, for CRC detection. This test targets the methylation abnormalities of *BMP3* and *NDRG4* alongside seven site mutations of *KRAS* [89,90]. *BMP3* is a member of the transforming growth factor (TGF) superfamily that plays a crucial role in embryonic development by initiating and patterning the creation of the early skeleton. It is reported that *BMP3* regulated colon tumorigenesis through an ActRIIB/SMAD2-dependent and TAK1/JNK signaling pathway [91]. According to one study, *BMP3* is hypermethylated in CRC, which is detrimental since it inhibits its function [92]. *NDRG4* contributes to cell proliferation and differentiation, and its expression is reduced in CRC [93].

Aside from *SEPT9* and the combination of *NDRG4* and *BMP3*, another commercial screening and diagnostic method based on the Heparan sulfate proteoglycan syndecan-2 protein (SDC2) was developed. SDC2 is a receptor for extracellular matrix elements on the cell surface [94]. SDC2 upregulation in CRC is highly associated with vascular invasion, cancer stage, and metastasis [95]. Early-tect and Colosafe are SDC2 detection kits developed in South Korea and China, respectively [96,97]. Methylated SDC2 demonstrates a sensitivity ranging from 77.0% to 93.9% and a specificity ranging from 97.4% to 98.1% for all stages of CRC screening utilizing stool samples [98–100]. Another gene known to have the potential to be a biomarker is the vimentin gene (*VIM*) [101,102]. Normal mesenchymal cells express *VIM*, which codes for the intermediate filament protein involved in cellular structure and stability [103]. Vimentin influences the proliferation, invasion, and migration of CRC via regulated activator protein 1 (AP-1) [104]. Aberrant methylation of exon-1 regions within the non-transcribed *VIM* can be successfully detected in fecal DNA to identify approximately half of patients with CRC with sensitivity of 46% and specificity of 90% [105]. By activating the focal adhesion signaling pathway, *FSTL1* interacts with *VIM* and promotes CRC metastasis [106]. Other reported methylated genes also include *SFRP1* [76,107], *SFRP2* [76,107], *DKK2* [107], *NEUROG1* [108], *SEPT7*, and *ALX4* [109]. These studies show that DNA methylation might serve as an undeniable potential to detect and diagnose CRC in the near future.

### Histone modification shows potential as an indicator in CRC detection

Histone proteins are important chromatin components that wrap DNA into nucleosomes and fold it into higher-order structures [68]. Histone modifications are most frequently seen in these four histones: H2A, H2B, H3, and H4. These histones are arranged in cylinder-like structures and comprise the histone core [73]. Histone modifications in localized promoter regions, including phosphorylation, acetylation, or methylation, are histone codes for chromatin packing and transcription [110]. Numerous studies highlight the significant role of histone modification in the development of CRC [111], indicating its potential as a biomarker for the disease [112–114]. The two histone aberrations most frequently studied in CRC are histone acetylation and methylation [69,115].

CRC and adenomas have significantly elevated levels of H3K9 methylation compared to normal colonic mucosa, but CRC is characterized by increased acetylation levels at H3K27 and H4K12 compared to normal colonic mucosa [116–118]. The stability of these modifications in circulation has prompted research into their utility for cancer detection. Patients with CRC had significantly lower levels of H3K9me3 and H4K20me3 in circulating nucleosomes, as determined by chromatin immunoprecipitation, compared to healthy individuals [119]. Other preliminary investigations utilizing ELISA-based assays indicated that H3K27me3 and H4K20me3 levels in patients with CRC were considerably lower than in individuals without cancer [120]. The histone methyltransferase WHSC1, a histone methyltransferase, facilitates dimethylation of H3K36me2, which is highly expressed in CRC via targeting anti-apoptotic *BCL2* [121]. Although histone modification is less popular than other epigenetic modifications, its potential value for diagnostic and CRC screening is promising.

### The role of miRNA as a novel biomarker in CRC diagnosis and screening

miRNA, a type of small non-coding RNA (sncRNA), typically ranges from 18 to 25 nucleotides in length [122]. By causing the breakdown of mRNAs or preventing translation, miRNAs can control the translation of target genes [123]. These extracellular miRNAs functioning as signaling molecules facilitating cell-to-cell communication can be detected in serum and bodily fluids, making them potent biomarkers [124]. miRNA can exist stably in body fluids like serum or blood plasma, associated with lipid-based carriers such as lipoprotein [125,126]. In addition to blood samples, miRNA can also be found in feces as colonocytes exfoliate and shed into the lumen of the gastrointestinal tract regularly [127,128]. miRNAs have numerous cellular functions closely related to cancer development, such as cell proliferation, migration, differentiation, and apoptosis [129,130]. Multiple reports have shown significantly different expression of miRNAs between patients with CRC and healthy individuals [68,131,132].

Several miRNAs have been identified in CRC tissue samples, including miR-21, miR-17, miR-20a, and miR-32 [133]. Reports showed that miR-21, which is upregulated in CRC, is one of the most highlighted oncomiRs in CRC [68,123,134]. miR-21 has several functions in cell biology, such as cell proliferation, adhesion, angiogenesis, migration, invasion, metabolism, and anti-apoptosis [132]. Increased levels of miR-21, miR-29a, and miR125b in serum could discriminate patients with early colorectal neoplasms, and the increase in serum miR125b levels might represent an early phase of colorectal carcinogenesis [135]. miRNA-21 and miRNA-200b are frequently upregulated in CRC cells [68]. Correlations were observed between miR-21 levels and matched tissue expression levels, reinforcing its potential as a significant indicator [70,134]. Additionally, the levels of miR-21 in the serum made a clear distinction between patients with adenoma and CRC [136]. According to a study utilizing a panel consisting of miR21, miR25, miR18a, and miR22, only miR21 concentrations exhibited a significant increase three years before diagnosis, suggesting its diagnostic utility [137]. In addition, miRNA markers may serve as important tools in prognostication. A study demonstrated that elevated levels of the microRNA miR-141 in plasma were associated with poor prognosis [138]. Another study yielded different results depending on whether free circulating or exosomal miRNA was measured. There was no discernible difference in the levels of miRNA found in the serum. However, exosomal levels of miR-16, miR-23, and let-7 were different between patients with CRC and controls [139]. It was reported that the loss of tumor-suppressing miRNAs, also known as anti-oncomiRs, during reduced global miRNA had a greater impact on promoting carcinogenesis than the loss of oncogenic miRNAs (oncomiRs) [123]. Recent studies have identified several anti-oncomiRs, including miR-181b [140], Let7 [141], miR29b [142], and miR145 [143].

Research on miRNA in the field of cancer is still in its early stages, presenting numerous challenges that need to be addressed. While it has been proposed that stool-based miRNA could be used for CRC screening, there are concerns due to the presence of DNA and RNA from gut microbiota in stool, making it uncertain if this is the optimal screening method [144]. It may be possible to improve detection accuracy by using both FIT and stool-based miRNA markers to address this issue. According to a previous investigation, using miRNA in conjunction with FIT improved the efficacy of fecal-based FIT on its own [145]. Combining miR-21 and miR-92a with other screening strategies, such as FIT, increased the specificity to 96.8% and the sensitivity to 78.4% from 98.4% and 66.7%, respectively [127].

Another disadvantage associated with miRNA is the lack of organ specificity observed in its expression. This is a common issue with many miRNA markers, as their dysregulation often overlaps with various cancer types. For example, miR-21 was found to have significant expression levels in patients diagnosed with lung, breast, esophageal, and gastric malignancies [146]. Because a single diagnostic marker would only cover one disease pathway, using multiple biomarkers could improve miRNA sensitivity and specificity, as demonstrated in a study involving miRNA-1246, miRNA-202-3p, miRNA-21-3p, miRNA-1229-3p, and miRNA-532-3p. According to this study, the panel combination had 91.6% sensitivity and 91.7% specificity in differentiating CRC from healthy individuals and 94.4% sensitivity and 84.7% specificity in distinguishing CRC from adenoma [147]. As research advances, a growing body of knowledge on the role and potential of miRNAs continues to emerge, and it is increasingly likely that a biomarker panel suitable for detecting CRC could be established.

### Expert commentary

In the past decades, many efforts have been made to decrease cancer incidence and improve survival rates. One of the important approaches has been the development of effective, feasible, and minimally invasive screening and diagnostic tools. Colonoscopy, the current golden standard for CRC detection, is invasive and requires bowel preparation. CRC diagnosis and screening were transformed when occult blood in stool testing was introduced. This method has improved over the years and has become more accurate, but it still has some drawbacks (Table 1). For instance, it is only able to detect CRC that originated as a result of bleeding lesions, which is something that might happen at random, whereas colorectal cancer can develop and progress even in the absence of bleeding. Much work has recently been put into enhancing CRC screening, including discovering genetic and epigenetic biomarkers. These biomarkers have allowed for earlier detection of the disease. Despite significant progress in genetic and epigenetic CRC-related research, their usage is still limited since the screening and diagnostic capability of the vast majority of genetic and epigenetic markers vary, rarely give diagnostically conclusive information, and only a few have been approved to be used in clinical settings (Table 1). In addition, some of the epigenetic changes observed in CRC were also found in other solid tumors outside CRC. A single biomarker appropriate for all CRC symptoms is difficult to find because of the significant molecular heterogeneity of CRC, making it challenging to determine which method is superior. Various studies have indicated that a combination of diagnostic techniques can increase sensitivity. This raises questions about whether genetic and epigenetic biomarkers can serve as standalone screening tests multi-gene biomarkers, or if they should be combined with other tests like gFOBT or FIT. Other considerations include the frequency of testing for high-risk patients. Consequently, a systematic evaluation of available tests and clinical studies is essential to determine the optimal screening approach for patients. Compared to other markers, DNA methylation is better understood in terms of potential as a screening and diagnostic biomarker. While many biomarkers are being researched, only a few are recommended for clinical use.

**Table 1:** List of genetic and epigenetic biomarkers candidates for screening and early detection of CRC

Categories	Gene	Sample	Evidence**	Commentary
**Genetic**				
	*BRAF* [148–150]	Tissue, blood	High	•Strongly associated with gene co-methylation •Usually used for distinguishing familial MSI-High CRC from sporadic CRC •Reported as therapeutical predictive markers (unresponsive to anti-EGFR) •In serum sample shows variable results
	*KRAS* [148,151], *NRAS* [152], *APC* [153], *MMR* genes (*MLH1*, *MSH2*, *MSH6*, *PMS2*, *EPCAM*) [154]	Tissue	High	•Reported as therapeutical predictive markers (unresponsive to anti-EGFR) •Associated with prognostic status
	*PTEN* [155], *STK11* [156], *CSMD1* [157], *PIK3CA* [156]	Tissue	Low	•Studies showed variable results •Low occurrence of CRC
**Epigenetic**				
**Single DNA** **methylation marker**	*SEPT9* [85,158,159]	Stool, blood	High	•The most studied epigenetic marker with a large number of samples and studies. •FDA-approved and has been used in clinical settings
	Vimentin [103,148], *SDC2* [160–162]	Stool, blood	Moderate	•The value of this gene differs depending on the samples •A moderate number of studies and samples •A low number of studies with paired samples •Stool Vimentin could detect adenoma while there is no data regarding blood sample
	*EYA2* [148], *GATA4* [103], *IGFBP3* [163], *NDRG4* [103], *NEUROG1* [108], *SFRP2* [103], *TFPI2* [164], *WIF1* [165], *ALX4* [148]*	Stool, blood	Low	•Small number sample size •Small number of studies
**Panel DNA** **methylation markers**	*NDRG4*, *BMP3*, *KRAS* mutation (genetic), hemoglobin [166–169]	Stool, blood	High	•A large number of studies and sample size •High sensitivity and specificity •Used in clinical settings •FDA approved
	*ALX4*, *BMP3*, *NPTX2*, *RARB*, *SDC2*, *SEPT9*, and *VIM* [170], *SFRP2*, *GATA4/5*, *NDRG4* and *VIM* [103], *ITGA4*, *SFRP2*, and *p16* [171], *SEPT9* and *ALX4* [109], *SFRP1*, *SFRP1*, *TFP12*, and *IKZF1* [172], *IRF4*, *IKZF1* and *BCAT1* [173], *IGFBP3* and *miR137* [163], *IGFBP3* and *TWIST1*[163], *SEPT9* and *ALX4* [109], *SFRP2*, *TFPI2*, *NDRG4*, and *BMP3* [174], *ALX4*, *SEPT9*, and TMEFF2 [175], *APC*, *MGMT*, *RASSF2A*, and *WIF1* [176], *BCAT1* and *IKZF1* [177], *TFPI2* and *SDC2* [178]	Stool, blood	Low	•A small number of studies •Varied results
**Histone modifications**	H3K9me3 [118,179], H4K20me3 [179]	Tissue	Low	•A small number of samples •A small number of studies •A primary tissue sample is not convenient compared to blood or stool samples
**Single miRNA marker**	miR-21 [180-183]*, miR-92a [182]*, miR-29a [183]*, miR20a, miR106a [182],miR223, miR-143/miR145 [182]*,miR221, miR135b [183]*, miR31 [182]	Stool, blood	Low	•Varied results •Low to moderate sensitivity •Can be upregulated in other malignancies
**Panel miRNA markers**	miR-21, miR-29a, and miR-125b [183], miR-21, let-7g, miR-31, miR- 92a, miR-181b, and miR-203 [184], miR-601, miR-760*[185], miR-29a and 92a*[186], miR-532-3p, miR-331, miR- 195, miR-17, miR-142-3p, miR-15b, miR-532, and miR- 652 *[187], miR-19a-3p, miR-223-3p, miR-92a-3p and miR-422a [188]*	Blood	Low	•Varied results •A small number of studies •Low to moderate sensitivity •Can be upregulated in other malignancies
	miR-223 and miR-92a [189], miR-21 and miR-92a*[190]	Stool, blood	Low	•A small number of studies •Low to moderate sensitivity •Can be upregulated in other malignancies

* Including for detecting adenoma

** Evidence is based on number of studies, number of sample used in studies, and whether the biomarkers have been used in clinical settings

## CONCLUSION

Growing evidence indicates that genetic and epigenetic circulating biomarkers have significant potential for noninvasive screening and diagnosing patients with CRC. Although substantial advancements have been made, numerous challenges remain to be addressed before these biomarkers can be effectively applied in clinical settings. One of them is numerous pathway disruptions resulting from CRC heterogeneity. To address this challenge, a promising approach involves the utilization of a panel of biomarkers rather than relying on a single biomarker. Alternatively, combining these biomarkers with existing methods, such as FIT, could enhance sensitivity and specificity, thereby circumventing the limitations posed by CRC heterogeneity. Large-scale multi-center trials involving diverse populations are needed for future clinical applications.

## Personal thanks

We thank the Directorate of Research and Community Service, Minister of Education, Culture, Research, and Technology of Indonesia.
